# The 20-item prosopagnosia index (PI20): a self-report instrument for identifying developmental prosopagnosia

**DOI:** 10.1098/rsos.140343

**Published:** 2015-06-24

**Authors:** Punit Shah, Anne Gaule, Sophie Sowden, Geoffrey Bird, Richard Cook

**Affiliations:** 1 Social, Genetic and Developmental Psychiatry Centre (MRC), Institute of Psychiatry, Psychology and Neuroscience, King's College London, University of London, London, UK; 2 Department of Psychology, City University London, London, UK; 3 Division of Psychology and Language Sciences, University College London, Bedford Way, London, UK; 4 Institute of Cognitive Neuroscience, University College London, London, UK

**Keywords:** developmental prosopagnosia, self-report, questionnaire, face blindness, congenital prosopagnosia, face perception

## Abstract

Self-report plays a key role in the identification of developmental prosopagnosia (DP), providing complementary evidence to computer-based tests of face recognition ability, aiding interpretation of scores. However, the lack of standardized self-report instruments has contributed to heterogeneous reporting standards for self-report evidence in DP research. The lack of standardization prevents comparison across samples and limits investigation of the relationship between objective tests of face processing and self-report measures. To address these issues, this paper introduces the PI20; a 20-item self-report measure for quantifying prosopagnosic traits. The new instrument successfully distinguishes suspected prosopagnosics from typically developed adults. Strong correlations were also observed between PI20 scores and performance on objective tests of familiar and unfamiliar face recognition ability, confirming that people have the necessary insight into their own face recognition ability required by a self-report instrument. Importantly, PI20 scores did not correlate with recognition of non-face objects, indicating that the instrument measures face recognition, and not a general perceptual impairment. These results suggest that the PI20 can play a valuable role in identifying DP. A freely available self-report instrument will permit more effective description of self-report diagnostic evidence, thereby facilitating greater comparison of prosopagnosic samples, and more reliable classification.

## 1. Introduction

Developmental prosopagnosia^1^ (DP) is a neurodevelopmental condition characterized by face recognition difficulties, despite normal intelligence, typical visual acuity and intact socio-cognitive abilities [1–6]. Unlike cases of acquired prosopagnosia [7], deficits are seen in the absence of brain injury. In the majority of cases, ability to judge facial emotion [8,9], and make other social attributions [10,11], is unaffected. DP often runs in families, suggestive of a genetic component [12–15]. Individuals with DP often avoid social situations, experiencing feelings of guilt and shame about actual or imagined offence caused to others [16]. Long-term consequences can include a reduced social circle, loss of self-confidence and limited work opportunities [17–19].

Studies have estimated that DP may affect between 1.9% [20] and 2.5% [21] of the population. Crucially, however, identifying cases is not straightforward [22]: DP is not listed in the *Diagnostic and Statistical Manual of Mental Disorders* (DSM-5 [23]) as a psychiatric disorder and no formal diagnostic criteria exist. Current approaches to ‘diagnosis’ emphasize performance on objective, computer-based tests of face recognition ability, including the Benton Facial Recognition Test [24], different versions of the Famous Faces Recognition Test (FFRT [25]), the Cambridge Face Memory Test (CFMT [26]) and the Cambridge Face Perception Test (CFPT [13]). The current consensus is that an individual should demonstrate substantial impairment (e.g. a score of 2 s.d. below the mean of a matched control sample) on either the CFPT or the CFMT—regarded as the leading objective tests of face recognition—to be diagnosed with DP [27].

Few would disagree that objective tests have been crucial for establishing our current knowledge of DP and must continue to play a key role in identifying individuals with the disorder [4,27]. However, relying exclusively on these tasks for diagnostic evidence may not always produce reliable classification. Whether a given score falls within the prosopagnosic range—beyond 2 s.d. of the control mean—is sensitive to the composition of the control group, and may be affected by control-participants' age, gender, IQ and ethnic origin [27–29]. Equally, cut-offs are affected by the spread of control scores and the treatment of outliers. Further factors, unrelated to the presence of DP, may also cause someone to perform badly, including poor motivation, misinterpretation of instructions (e.g. prioritizing response speed over accuracy), poor mouse control and test anxiety [30]. For example, administering the CFMT and CFPT to undergraduate samples routinely produces scores within the prosopagnosic range [27], however the nature of these outlying scores remains unclear; many of the individuals identified report entirely normal face-recognition in their daily lives.

Conversely, individuals may have genuinely impaired face perception but perform within the normal range on computer-based tests. For example, prosopagnosics may develop compensatory strategies to negotiate laboratory tasks or have perceptual issues extending beyond static greyscale faces. Given the three-alternative-forced-choice (3AFC) format of the CFMT, some respondents will gain a non-trivial number of hits by chance. It is worth noting an asymmetry in the standard of evidence provided by the CFMT and related tests employing this format: whereas errors are unlikely to be due to chance (where correct responses are known, participants rarely enter incorrect responses erroneously), correct answers may often reflect lucky guesses (a third of guesses will be correct in a 3AFC task). Consequently, scores within the typical range may not always be strong evidence of intact perception.

In recognition of the foregoing issues, few researchers attempt to characterize DP using a single diagnostic instrument; rather, leading research groups use a number of tests to develop a perceptual profile, and diagnose DP only where convergent evidence accumulates. In practice, self-report forms a key part of this profile. When contacted by suspected prosopagnosics, researchers routinely conduct interviews (e.g. [31,32]) or administer bespoke questionnaires. This self-report evidence is valuable when interpreting performance on computer-based tasks. For example, a low score on a computer**-**based test in the absence of self-reported problems (or vice versa) may be regarded with caution and additional testing conducted. However, the absence of standardized instruments has led to poor reporting of self-report evidence. Articles are often vague with respect to the methods and results of self-report assessments. Moreover, the use of different instruments by different research groups has prevented comparison of self-reported problems across samples.

To help address these issues, we present a short, validated self-report questionnaire for assessing prosopagnosic traits. Self-report questionnaires have been crucial in the study of other neurodevelopmental disorders (e.g. [33]) and often prove significant in the development of formal diagnostic criteria [34]. The only existing self-report questionnaire for prosopagnosic traits [21] has been criticized on the grounds that it correlates poorly with objective measures of face recognition ability [35]. Published correlations between scores on this scale and objective tests of face recognition ability range from *r*=0.20 [15] to 0.55 [36]. The weak relationship observed probably reflects the inclusion of items pertaining to navigation deficits, the presence of face recognition difficulties in the respondents' wider family, and ability to judge facial attractiveness, facial emotion and facial gender: issues that are not reliable features of DP.^2^

## 2. The 20-item prosopagnosia index

The 20-item prosopagnosia index (PI20) is a self-report instrument assessing the presence of prosopagnosic traits. Respondents indicate the extent to which 20 statements describe their face recognition experiences (table 1). Agreement is scored on a five-point scale (strongly agree to strongly disagree). Fifteen statements are scored positively, whereby strongly agree is scored ‘5’ and strongly disagree is scored ‘1’. Five items are reverse scored (strongly agree is scored ‘1’ and strongly disagree is scored ‘5’).

**Table 1.** The 20 statements comprising the PI20, shown with the mean scores for the TD controls and suspected prosopagnosics for each item.

		controls	troubled
1	My face recognition ability is worse than most people	1.88 (*0.89*)	4.66 (*0.51*)
2	I have always had a bad memory for faces	1.88 (*0.96*)	4.55 (*0.74*)
3	I find it notably easier to recognize people who have distinctive facial features	3.69 (*1.11*)	4.31 (*1.00*)
4	I often mistake people I have met before for strangers	1.90 (*1.04*)	4.54 (*0.70*)
5	When I was at school I struggled to recognize my classmates	1.34 (*0.72*)	3.43 (*1.25*)
6	When people change their hairstyle, or wear hats, I have problems recognizing them	1.86 (*0.95*)	4.33 (*0.86*)
7	I sometimes have to warn new people I meet that I am ‘bad with faces’	1.47 (*0.85*)	4.12 (*1.07*)
8*	I find it easy to picture individual faces in my mind	2.38 (*1.16*)	4.25 (*0.96*)
9*	I am better than most people at putting a ‘name to a face’	2.76 (*1.17*)	4.55 (*0.80*)
10	Without hearing people's voices, I struggle to recognize them	1.66 (*0.87*)	3.78 (*1.03*)
11	Anxiety about face recognition has led me to avoid certain social or professional situations	1.36 (*0.79*)	3.76 (*1.27*)
12	I have to try harder than other people to memorize faces	1.84 (*1.03*)	4.43 (*0.76*)
13*	I am very confident in my ability to recognize myself in photographs	1.42 (*0.87*)	2.58 (*1.28*)
14	I sometimes find movies hard to follow because of difficulties recognizing characters	1.73 (*1.08*)	4.52 (*0.75*)
15	My friends and family think I have bad face recognition or bad face memory	1.50 (*0.87*)	4.16 (*0.83*)
16	I feel like I frequently offend people by not recognizing who they are	1.69 (*0.98*)	4.25 (*0.79*)
17*	It is easy for me to recognize individuals in situations that require people to wear similar clothes (e.g. suits, uniforms and swimwear)	2.55 (*1.24*)	4.25 (*1.05*)
18	At family gatherings, I sometimes confuse individual family members	1.36 (*0.83*)	2.67 (*1.25*)
19*	I find it easy to recognize celebrities in ‘before-they-were-famous’ photos, even if they have changed considerably	2.79 (*1.23*)	4.61 (*0.72*)
20	It is hard to recognize familiar people when I meet them out of context (e.g. meeting a work colleague unexpectedly while shopping)	2.01 (*1.11*)	4.49 (*0.66*)

Standard deviations are shown in italics inside parentheses. Items marked with asterisks (*) are reverse scored.

Items were generated following review of the qualitative [16,18,19,37–39] and quantitative literature (e.g. [1,2]) on DP, and through discussions with DPs. Items require no previous knowledge about DP, permitting the identification of sufferers who are unaware they have the condition. No items relate to emotion recognition, navigation difficulties, problems judging facial attractiveness and facial gender. Furthermore, no items were included on the presence of DP in the respondents' wider family, ensuring that the PI20 can be used in quantitative genetic studies estimating the heritability of the condition (defining DP using such criteria renders any conclusion about heritability circular).

## 3. Validation Study 1

The PI20 purports to measure prosopagnosic traits. A key indicator of its construct validity is therefore its ability to distinguish known or suspected DPs from the wider population. To determine whether the PI20 satisfies this fundamental criterion, the questionnaire was administered remotely via the Internet, to a sample of suspected DPs and typically developed (TD) controls.

### 3.1 Participants and methods

Three-hundred-and-nineteen adults aged between 18 and 74 years participated in Validation Study 1: 242 TD (*M*_age_=29.8 years; 87 males) and 77 suspected DPs (*M*_age_=43.0 years; 30 males). All participants reported normal or corrected-to-normal vision. TD participants were recruited using a local participant database. Suspected DPs contacted the authors via www.troublewithfaces.org complaining of face recognition difficulties, or were recruited via online communities for individuals with DP. Importantly, these individuals identified themselves as suspected prosopagnosics *before* administration of the PI20 questionnaire. Typically, the suspected DPs had heard about the condition through friends, family or the popular media. Having sought further information from a variety of sources, and recognized the features and anecdotes described, individuals made themselves known to the authors.

### 3.2 Results

**Figure 1.**
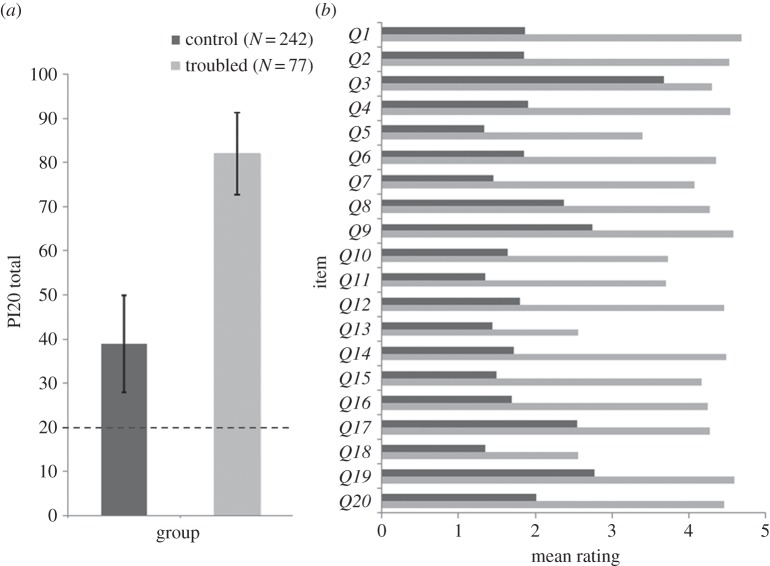
(*a*) Mean PI20 totals observed for the suspected prosopagnosics and TD controls in Validation Study 1. Error bars represent ±1 s.d. The dashed line at 20 represents the minimum possible score. (*b*) Mean responses given by the suspected prosopagnosics and TD controls for each item.

The mean PI20 score of the suspected DPs (*M*=82.01, s.d.=9.34; figure 1) far exceeded that of the controls (*M*=38.90, s.d.=10.88), *t*_317_=31.29, *p*<0.001. Of the 77 suspected DPs, 74 (96.1%) scored more than 2.5 s.d. above the control mean (more than or equal to 66), and 67 (87.0%) scored more than 3 s.d. above the control mean (more than or equal to 72). The mean responses given by the two samples differed significantly on all 20 items (all *t*'s>4.47, all *p*'s<0.001). The 319 responses yielded a Chronbach's *α* of 0.96, indicating that the 20 items have high internal consistency. Exploratory factor analysis with Varimax rotation also suggested a strong single factor structure accounting for 61% of the variance in responses.^3^

## 4. Validation Study 2

Many of the respondents described in Validation study 1 have never undergone formal testing of their face recognition ability. It is therefore likely that both groups are heterogeneous: some self-diagnosed DPs may in fact fall within the typical range of face recognition ability and some of the individuals identified as TD may exhibit some prosopagnosic traits. Validation Study 2 therefore sought to confirm that the PI20 distinguishes between prosopagnosics whose deficits have been verified by formal testing and age-matched controls.

### 4.1 Participants and methods

Thirty-six participants aged between 20 and 74 years completed Validation Study 2: 18 suspected prosopagnosics (*M*_age_=46.7; 12 males), recruited via www.troublewithfaces.org, and 18 matched TD controls (*M*_age_=43.5; 12 males), recruited via the local participant database. All participants completed a range of computer-based tests in our laboratory facilities to assess their face and object recognition ability, including the CFMT [26], the CFPT [13], a version of the FFRT [25] and the CCMT [40]. The scores of the DP group and the control group are shown in table 2. Comparing performance on this battery of tasks is commonly used to diagnose DP. All members of the DP group exhibited evidence of impairment on convergent objective tests of face recognition ability.

**Table 2.** The scores achieved on PI20 and other diagnostic tests by each member of the prosopagnosic sample. Scores on the Famous Faces Recognition Test (FFRT), the Cambridge Face Memory Test (CFMT) and Cambridge Car Memory Test (CCMT) reflect % correct. Scores on the Cambridge Face Perception Test (CFPT) reflect total deviation errors.

case	age	gender	PI20	FFRT (%)	CFMT (%)	CCMT (%)	CFPT upright	CFPT inverted
1	20	M	84***	52*	60**	82	50*	60
2	24	M	73**	59	60**	50*	60**	78
3	41	F	73**	73	68*	85	48*	64
4	31	M	71**	31**	56**	65	30	70
5	33	F	87***	46*	60**	74	78***	84*
6	36	M	87***	24**	57**	56*	42*	60
7	42	M	78***	44*	58**	93	52**	50
8	45	M	92***	15***	51***	94	86***	54
9	48	F	78***	30**	58**	86	34	52
10	51	F	85***	42*	46***	64	74***	94*
11	57	M	69**	48*	61**	53*	32	52
12	59	M	97***	3***	49***	82	56**	64
13	67	M	92***	10***	28***	47**	92***	78
14	69	F	95***	32**	36***	76	100***	92*
15	74	M	82***	34**	60**	67	42*	70
16	73	M	68**	30**	53***	72	84***	90*
17	42	M	83***	27**	56**	90	44*	100**
18	29	F	68**	48*	61**	64	32	58
control mean			41.7	74.9	84.3	77.2	29.4	63.3
control s.d.			12.1	17.5	9.9	15.0	10.9	15.6
best control			23	100	99	100	10	36
worst control			65	34	67	47	52	84

Asterisks denote: single (*), differs from control mean 1 s.d.; double (**), differs from control mean 2 s.d.; triple (***), differs from control mean 3 s.d.

### 4.2 Results and discussion

The mean PI20 score of the suspected DPs (*M*=81.22, s.d.=9.47) again exceeded that of the controls (*M*=41.67, s.d.=12.10), *t*_34_=10.92, *p*<0.001. Of the 18 suspected DPs, 13 scored more than 2.5 s.d. above the control mean (more than or equal to 72), and 12 scored more than 3 s.d. above the control mean (more than or equal to 78). The mean PI20 score of the DP and TD groups in Validation Study 1 and 2 corresponded closely. Importantly, these results confirm that the PI20 distinguishes between prosopagnosics whose deficits have been verified by formal testing and age-matched controls. The results of the Validation Studies 1 and 2 indicate that the PI20 measures the DP construct as it is currently understood. Suspected DPs recognize the experiences and anecdotes contained within the PI20 and the resulting scores afford classification convergent with existing diagnostic procedures. In Validation Studies 3–5, we assessed the relationship between PI20 scores and objective measures of face and object recognition in more detail.

## 5. Validation Study 3

In our third validation study, we sought to determine whether PI20 scores correlate with respondents' ability to recognize famous faces. Any suggestion that self-report measures can contribute to the classification of DP rests on the assumption that people have insight into their own face recognition ability. If respondents are poor judges of their face recognition ability, high self-report scores may simply reflect respondents' personality; for example, some individuals are known to underestimate their cognitive abilities [41]. Alternatively, strong correlation between PI20 scores and objective measures of face recognition ability would confirm that individuals *do* have insight into their face recognition ability.

### 5.1 Participants and methods

One-hundred-and-seventy-three of the respondents from Validation Study 1, aged between 18 and 74 years, including 100 TD (*M*_age_=30.0; 27 males; 84 UK-based) and 73 suspected DPs (*M*_age_=42.9; 28 males; 39 UK-based) participated in Validation Study 3. This sample included participants from Validation Study 2 (see the electronic supplementary material). All participants completed an Internet-based version of the FFRT [25] remotely, during which they had to identify 34 international celebrities (actors, singers, sports stars and politicians), from cropped photographic images, by providing their name or other identifying information. Faces were visible until participants responded. Scores reflect the number of correct identifications expressed as a percentage of the number of celebrities with whom respondents were familiar.

### 5.2 Results

**Figure 2.**
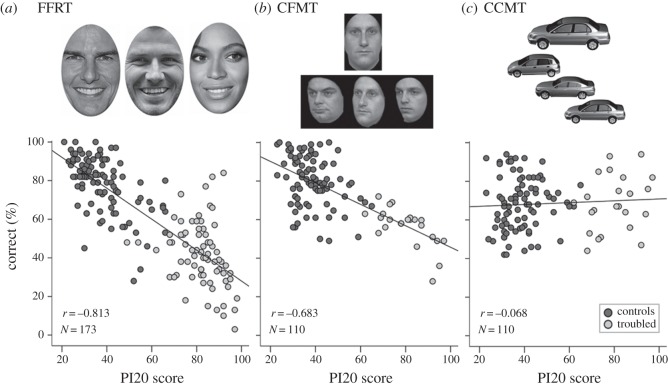
(*a*) The simple correlation observed between PI20 scores and performance on the FFRT in Validation Study 3. (*b*) The simple correlation observed between PI20 scores and performance on the CFMT observed in Validation Study 4. (*c*) The simple correlation observed between PI20 scores and performance on the CCMT observed in Validation Study 5.

The suspected DPs identified significantly fewer celebrities (*M*=41.73%, s.d.=16.58%) than controls (*M*=78.28%, s.d.=14.51%), *t*_171_=15.41, *p*<0.001. Importantly, PI20 scores correlated closely with participants' famous face recognition (*r*=−0.81, *p*<0.001; figure 2*a*). To control for the influence of demographic factors, participant age (years), gender (1=male; 2=female) and location (1=based in UK, 2=based outside UK), were entered into the first step of a hierarchical regression, with PI20 scores entered second. Participant age (*β*=−0.42, *p*<0.001), gender (*β*=0.16, *p*=0.021) and location (*β*=−0.17, *p*=0.015) were all significant predictors, together accounting for 28.8% of the variance. Crucially, PI20 score remained highly predictive (*β*=−0.76, *p*<0.001), accounting for a further 40.8% of unique variance. These results indicate that PI20 scores correlate with ability to recognize familiar faces.

## 6. Validation Study 4

Next, we sought to determine whether PI20 scores predict performance on the CFMT [26]. Whereas the FFRT used in Validation Study 3 assesses ability to recognize familiar faces, the CFMT measures ability to match unfamiliar faces, thought to depend on different neurocognitive mechanisms [42–44]. A correlation between PI20 scores and CFMT performance would confirm that respondents have insight into both their familiar and unfamiliar face recognition ability.

### 6.1 Participants and methods

One-hundred-and-ten participants from Validation Study 1, aged between 18 and 74 years, including 87 TD (*M*_age_=28.6 years; 30 males) and 23 suspected prosopagnosics (*M*_age_=45.8 years; 15 males) participated in Validation Study 4. A subset of the sample also participated in Validation Studies 2 and 3 (see the electronic supplementary material). All participants were living in the UK at the time of testing. The DP sample contacted the authors via www.troublewithfaces.org. TD participants were recruited through the local participant database. All participants completed the CFMT in our laboratory facilities. The test comprises 72 trials and employs a 3AFC match-to-sample design. Participants first learn a target face in left three-quarters-profile view, frontal view and right three-quarters-profile view. During a subsequent recall phase, participants are required to identify the target in a 3AFC procedure [26].

### 6.2 Results

The suspected DPs were significantly impaired on the CFMT (*M*=56.42%, s.d.=10.04%) relative to controls (*M*=79.98%, s.d.=13.00%), *t*_108_=8.07, *p*<0.001. Crucially, PI20 scores correlated closely with participants' CFMT scores (*r*=−0.68, *p*<0.001; figure 2*b*). Additional hierarchical regression analysis was conducted to control for the influence of participant age (years) and gender (1=male; 2=female). When entered in the first step of the model, participant age was predictive (*β*=−0.40, *p*<0.001), but participant gender was not predictive (*β*=0.10, *p*=0.30), of CFMT performance. Together, these factors accounted for 19.1% of the variance. Importantly, when added to the model, PI20 scores remained highly predictive of CFMT score (*β*=−0.65, *p*<0.001), accounting for a further 27.8% of unique variance. The results of Validation Study 3 indicate that PI20 scores correlate with unfamiliar face recognition. Together, Validation Studies 3 and 4 confirm that people have insight into their face recognition ability.

## 7. Validation Study 5

The results of Validation Studies 1–4 confirm that PI20 scores correlate with familiar and unfamiliar face recognition. One account of this relationship is that the PI20 measures a relatively specific construct—face recognition ability. However, a second possibility is that the PI20 measures a broader construct (e.g. general memory ability). If the PI20 is measuring a general factor, scores should also correlate with performance on the Cambridge Car Memory Test (CCMT [40]), a well-validated test of non-face object recognition employing an identical format to the CFMT.

### 7.1 Participants, methods and results

The CCMT was administered to the 110 respondents who participated in Validation Study 4. The car recognition ability of the suspected DPs (*M*=68.64%, s.d.=14.34%) was very similar to that of the TD controls (*M*=68.29%, s.d.=13.56%), *t*_108_=0.11, *p*=0.91. No correlation was observed between participants' PI20 scores and performance on the CCMT (*r*=0.07; figure 2*c*). Hierarchical regression was conducted to determine whether PI20 scores were predictive of CCMT performance once individual differences in age and gender were controlled for. When entered in the first step, participant age was not predictive of CCMT scores (*β*=0.08, *p*=0.44), but, in line with previous findings [40], respondent gender was a significant predictor (*β*=−0.20, *p*=0.044), together accounting for 5.6% of the variance. When subsequently added to the regression model, PI20 scores were not predictive (*β*=−0.04, *p*=0.71), accounting for a further 0.1% of unique variance. These results suggest that the PI20 is measuring face recognition ability, and not a general factor.

## 8. Discussion

This paper introduces the PI20, a 20-item self-report measure of prosopagnosic traits. The new instrument successfully distinguishes suspected DPs from TD adults (Validation Studies 1 and 2). Strong correlations were observed between PI20 scores and performance on objective tests of familiar (Validation Study 3) and unfamiliar face recognition ability (Validation Study 4). Importantly, PI20 scores do not correlate with non-face object recognition (Validation Study 5), indicating that the instrument measures face recognition ability, not a general factor (e.g. wider memory ability).

The results of Validation Studies 3 and 4 confirm that people have the necessary insight into their face recognition ability, required by a self-report instrument. These findings contradict previous suggestions that adults lack insight into their own face recognition ability [27,35]. For example, having asked undergraduates to rate their ability to recognize faces in everyday life ‘compared with the average person’, Bowles *et al*. [27] found only weak correlations between self-rated ability and performance on the CFMT and CFPT. However, ratings derived from a single question are likely to provide noisy estimates, making weak correlations unsurprising. In addition, items asking about tangible experiences, such as those included in the PI20 (e.g. ‘I sometimes find movies hard to follow because of difficulties recognizing characters’), may be less ambiguous than abstract questions about average face recognition ability.

The foregoing results suggest that the PI20 can play a valuable role in the identification of DP, permitting better description of self-report evidence, thereby facilitating greater comparison of prosopagnosic samples. Based on the relationships observed between PI20 scores and performance on objective tests of face recognition, PI20 scores in the ranges 65–74, 75–84, 85–100 may be broadly indicative of mild, moderate and severe DP, respectively. To be clear, we are not suggesting that the PI20 should replace objective tests of face recognition ability; rather we intend the PI20 to be used as a complementary diagnostic instrument. Where PI20 and computer-based tests provide convergent evidence of impairment, authors can be confident in the composition of prosopagnosic samples. Conversely, where there is discrepancy between objective and self-report evidence, further testing can be undertaken, for example, to determine whether an individual has a severe, lifelong perceptual impairment that they are unaware of, or whether they have simply under-performed on a given task. The use of convergent tasks and complementary paradigms is likely to result in more reliable classification [34].

DP is a heterogeneous condition [4,36,45,46]. The inclusion of self-report measures in diagnostic batteries guards against the possibility that new sub-groups of the DP population go undetected because current computer-based tests are insensitive to their characteristic deficits. For example, current tests require participants to judge static facial images. However, the faces we encounter outside of the laboratory are dynamic [47,48]. Should prosopagnosics exist who have selective problems processing facial motion [49], they may perform within the normal range on current diagnostic tests, despite experiencing face recognition difficulties in their daily lives.

There have been calls for a quick, easy-to-administer instrument for the purposes of screening populations for DP [38]. Computer-based tests are unsuitable for screening large populations. For example, batteries of computerized tasks frequently exceed 45 min in duration and require control groups for interpretation. These factors, together with the expertise and equipment required to administer computer-based tests, limit their clinical and practical utility [30]. Conversely, the PI20 can be completed very quickly in the absence of a computer, by clinicians (e.g. to clients or patients), employers (e.g. to prospective police or border control officers) and judiciary (to eyewitnesses, jurors), to screen populations for DP. Future research directly assessing the PI20's utility as a screening tool in applied contexts will prove informative.

In academic contexts, the instrument may be used both to identify individuals with DP for inclusion in research samples (e.g. screening undergraduate cohorts) and to exclude individuals exhibiting prosopagnosic traits from studies addressing normative face perception. As we have noted, the PI20 is also well suited for use in genetic studies estimating the heritability of the condition. In addition, the availability of a validated self-report measure also permits systematic investigation of the relationship between self-report and objective measures of face recognition ability. For example, future studies might try to better understand which observers are likely to over- or underestimate their actual face recognition ability. Longitudinal studies might also address whether this variability is systematically related to changes in face recognition ability over time.

An increasing number of authors are taking advantage of online platforms to collect behavioural data remotely. While these methods facilitate the collection of large datasets, this trend has provoked considerable discussion about the quality of the data collected [50]. Some readers might therefore query our decision to collect the data reported in Validation Studies 1 and 3 online. A degree of caution is justifiable insofar as current understanding of research conducted online remains relatively limited and best practice continues to evolve. Importantly, however, the findings from these studies were replicated in Validation Studies 2 and 4, respectively, using controlled experimental procedures conducted in the laboratory. Not only do these results confirm that the PI20 can be administered effectively via the Internet—further underscoring its potential value as a screening instrument—but they support the view that online data collection has a valuable role to play in contemporary social perception research [51].

Discussion of the complementary roles played by self-report and objective tests in identification and diagnosis raises fundamental questions about the future of the DP construct. Although DP is not listed as a psychiatric disorder in DSM-5 [23], prosopagnosia is recognized by the World Health Organization [52], and potentially meets the criteria for a mental disability [53]; i.e. a physical or mental impairment that has a ‘substantial’ and ‘long-term’ negative effect on one's ability to do normal daily activities. Crucially, however, the extent to which DP impairs *normal daily activities* is not easily assessed with face recognition tests in the laboratory. Any attempt to move DP into mainstream psychiatry may therefore necessitate a broader approach to diagnosis, encompassing self-report.

## Supplementary Material

PI20 Questionnaire: Here we provide a version of the PI20 for download and use in research

## Supplementary Material

Validation data: This file contains the data collected during each of the validation studies.

## Footnotes

1 We use the term developmental prosopagnosia in preference to congenital prosopagnosia to reflect the possibility that the condition emerges during development, and may not necessarily be present from birth.

2 By including these items, the authors hoped to improve the specificity of the instrument; in other words, to exclude individuals from their DP research who report such problems as they are likely to suffer from a co-occurring condition [32]. However, this means that the instrument is not specifically designed for, nor is suitable for, the quantification of prosopagnosic traits.

3 The PI20 exhibited strong internal consistency when reliability analyses were restricted to the sample of suspected DPs (Chronbach's *α*=0.84). Similarly, a clear single factor structure was also present.

## Data accessibility

The dataset supporting this article are available as electronic supplementary material.
