# Supplementary material for: The 20-item prosopagnosia index (PI20): a self-report instrument for identifying developmental prosopagnosia
Source: R Soc Open Sci. 2015 Jun 24;2(6):140343. doi: 10.1098/rsos.140343 (PMC4632531; doi:10.1098/rsos.140343)
Supplement: PI20 Questionnaire: Here we provide a version of the PI20 for download and use in research [file rsos140343supp1.pdf]

troublewithfaces.org

The 20 - Item Prosopagnosia Index (PI20)

Punit Shah, Anne Gaule, Sophie Sowden, Geoff Bird, & Richard Cook

The following statements inquire about your face recognition abilities. For each item, indicate how much you agree or disagree by choosing the appropriate numbered response. Read each item carefully before responding and answer as honestly as possible. Thank you.

1	2	3	4	5
Strongly Disagree				Strongly Agree

1. My face recognition ability is worse than most people _____
2. I have always had a bad memory for faces _____
3. I find it noticeably easier to recognise people who have distinctive facial features _____
4. I often mistake people I have met before for strangers _____
5. When I was at school I struggled to recognise my classmates _____
6. When people change their hairstyle, or wear hats, I have problems recognising them _____
7. I sometimes have to warn new people I meet that I am ‘bad with faces’ _____
8. I find it easy to picture individual faces in my mind _____
9. I am better than most people at putting a ‘name to a face’ _____
10. Without hearing people’s voices I struggle to recognise them _____
11. Anxiety about face recognition has led me to avoid certain social or professional situations _____
12. I have to try harder than other people to memorise faces _____
13. I am very confident in my ability to recognise myself in photographs _____
14. I sometimes find movies hard to follow because of difficulties recognising characters _____
15. My friends and family think I have bad face recognition or bad face memory _____
16. I feel like I frequently offend people by not recognising who they are _____
17. It is easy for me to recognise individuals in situations that require people to wear similar clothes (e.g. suits, uniforms, swimwear) _____
18. At family gatherings I sometimes confuse individual family members _____
19. I find it easy to recognise celebrities in ‘before-they-were-famous’ photos, even if they have changed considerably _____
20. It is hard to recognise familiar people when I meet them out of context (e.g. meeting a work colleague unexpectedly while shopping) _____

Scoring

1. Items 8, 9, 13, 17 and 19 should be reverse scored. i.e., 5 = 1; 4 = 2; 3 = 3; 2 = 4; 1 = 5
2. Add together the numbered responses to calculate a score between 20 (unimpaired face recognition) - 100 (severely impaired face recognition)
